# Detection of Multiple Lineages of PRRSV in Breeding and Growing Swine Farms

**DOI:** 10.3389/fvets.2022.884733

**Published:** 2022-06-14

**Authors:** Ting-Yu Cheng, Magnus R. Campler, Declan C. Schroeder, My Yang, Sunil K. Mor, Juliana B. Ferreira, Andréia G. Arruda

**Affiliations:** ^1^Department of Veterinary Preventive Medicine, College of Veterinary Medicine, The Ohio State University, Columbus, OH, United States; ^2^Department of Veterinary Population Medicine, College of Veterinary Medicine, University of Minnesota, Saint Paul, MN, United States; ^3^School of Biological Sciences, University of Reading, Reading, United Kingdom; ^4^Department of Population Health & Pathobiology, College of Veterinary Medicine, North Carolina State University, Raleigh, NC, United States

**Keywords:** swine, porcine reproductive and respiratory syndrome virus, viral lineage, tonsil scrapings, oral fluid, processing fluid

## Abstract

The detection and co-circulation of multiple variants of porcine reproductive and respiratory syndrome virus (PRRSV) have been observed and reported in swine. However, the potential long-term impact of multiple prevailing PRRSV variants on pig-performance is not yet fully understood. The primary objective of this study was to describe the genetic variation of PRRSV in processing fluid (PF), oral fluid (OF), and tonsil scraping (TS) specimens from five swine farms with different production types and PRRS status over a period of time (~1 year). Furthermore, the association between PRRSV prevalence and production parameters was investigated. Results showed that PRRSV was detected by RT-qPCR in 21–25% of all types of specimens. In breeding farms, PRRSV detection in PF and/or TS samples was correlated with stillborn and mummified fetuses, and pre-weaning mortality throughout the study period. Although ORF5 sequences were obtained in <16% of all sample types, simultaneous detection of PRRSV variants including field and vaccine strains within a single sampling event was identified in both breeding and growing pig farms. Phylogenetic analyses based on the ORF5 sequence classified the detected field PRRSV into L1A and L1H, two sub-lineages of lineage 1 (L1). Our study demonstrated the presence of multiple PRRSV lineages, sub-lineages, and variants in swine herds and its potential association with swine reproductive performance under field conditions.

## Introduction

Since the first identification in the late 1980s, porcine reproductive and respiratory syndrome virus (PRRSV) has disseminated and become a major health concern in the global swine industry (1, 2). PRRSV has been recently classified as PRRSV-1 and PRRSV-2 species (*Betaarterivirus suid 1* and *Betaarterivirus suid 2*), which are commonly referred to as European and North American types, respectively (3). However, as a rapidly mutating RNA virus, various variants have been further reported in several countries including the U.S., China, and Spain (4–7). In 2013, PRRSV was estimated to result in a cost of ~$664 million annually in U.S. breeding and growing herds (8).

To detect PRRSV in swine herds, a variety of specimen types can be used. For several years, veterinarians relied on serum samples as the gold standard biological specimen for PRRSV detection (9). However, the use of aggregate specimens, e.g., oral fluid (OF) and processing fluid (PF) samples, has become more common because of their ease of collection, high sensitivity, and low cost (10–15). Recently, tonsil scraping (TS) has been described as a promising diagnostic specimen for growing pig populations (16). A recent study utilizing tonsil scraping samples for RT-PCR and bioassays has shown up to 77% detection rate (17), while another study found no difference among the efficacy of tonsil scrapings, blood, and oral fluids at 70-, 96- and 118-days post infection using RT-PCR (18).

Upon detection of PRRSV in the field, understanding the source of virus and ruling out re-emerging cases are common steps in PRRSV surveillance and PRRS outbreak investigations. Given the limited genetic information provided by RT-PCR, genetic characterization and variant differentiation are primarily done by sequencing the open reading frame 5 (ORF5) region of PRRSV (19). The ORF5 method has been further extended to predict how genetically related PRRSV isolates are, and to characterize existing diversity within a farm or system overtime. For example, a distinction of > 2–4% between a PRRSV ORF5 region sequenced before and after an outbreak is commonly interpreted as a new virus introduction. This concept has been utilized to investigate the genetic closeness of two virus isolates (20). More recently, lineage classification, an evolutionary approach to differentiate virus isolates, was adapted to PRRSV. Paploski et al. (21) classified 11,732 U.S. PRRSV-2 isolates (2001–2018) into eight lineages L1, L2, L4, L5–L9, and eight sub-lineages (L1A–L1H) within L1 (21). This classification system is currently being used to understand PRRSV diversity and discriminate between resident and newly introduced variants at the farm level.

Although the diversity of PRRSV has been described at national and regional levels (22–24), the co-circulation of multiple variants within sampling events of production sites was barely investigated, especially by collecting multiple samples for single time points throughout a long period of time (25). Furthermore, reports on the association between PRRSV detection and its potential impact on reproductive and growth performance are rare. Thus, the current study aimed to describe the genetic diversity of PRRSV detected in processing fluid (PF), oral fluid (OF), and tonsil scraping (TS) specimens from five U.S. swine farms (three farrow-to-wean, one wean-to-finish, and one finisher). The secondary aim was to investigate the association between PRRSV prevalence and swine production parameters of interest.

## Materials and Methods

### Farm Recruitment

Three farrow-to-wean (Farm 1: 5,000 sows; Farm 2: 6,000 sows; and Farm 3: 2,500 sows, respectively), one wean-to-finish (Farm 4; 3,550 pigs), and one finisher farm (Farm 5; 2,800 pigs) were recruited and categorized based on their PRRSV infection statuses, as previously published (25). Farm 1 had the latest PRRS outbreak ~6 months prior to the first sampling event and was considered as a “previously infected” herd, i.e., no ongoing PRRSV exposures (e.g., natural exposure, live virus inoculation, vaccination) during the study period. Farms 2 and 4 were infected <1 month prior to the first sampling and were classified as “recently infected herds” for the purposes of the study. In addition, a PRRSV lineage 8 modified-live vaccine (Fostera® PRRS, Zoetis Inc.) and a field PRRSV strain (undisclosable) were administered in Farm 2 within weeks of the recent outbreak, whereas no vaccination protocol was implemented in Farm 4. Farm 3 had no PRRSV outbreak history at recruitment but “actively vaccinated” the sow herd twice per year with a PRRSV lineage 5 modified live vaccine (Ingelvac PRRS® MLV, Boehringer Ingelheim). Likewise, although without PRRSV outbreak at recruitment, Farm 5 was classified as “actively vaccinated” as animals from that growing farm had received vaccination during the study period. In particular, the source sow farm vaccinated sows four times per year and piglets at processing (4–6 days of age) with the same modified live vaccine as Farm 3 due to an outbreak at the beginning of the study (previously described as farm 2 in [16]). PRRS-associated production indices were acquired from farm health records when possible. Production performance for breeding farms (Farm 1–3) was summarized using monthly average percentages of stillborn and mummified fetuses, and pre-weaning mortality in piglets. Likewise, for growing pigs, wean-to-finish (Farm 4) and grow-to-finish (Farm 5) mortalities and average daily weight gains (ADG) were collected. In this case, given these were available for the whole group of pigs, two production batches (approximate period of the study) for each of four barns in each farm were used.

### Sample Collection, RNA Extraction, and RT-qPCR

An Institutional Animal Care and Use Committee (IACUC) approval was not required for this project as all sample collections were performed by farm veterinarians for each participating farm. Processing fluids and pen-based oral fluid samples were collected from breeding (Farm 1–3) and growing pig (Farm 4–5) farms, respectively, while tonsil scrapings consisted of individual-based samples from all five farms. Each farm was sampled monthly by the farm veterinarian or directly instructed and trained farm personnel over a time period of ~1 year (February 2019–March 2020), i.e., with a goal of 12 sampling events per farm. At each monthly sampling event, PF samples (*n* = 8) were collected and aggregated from ≤ 20 processed litters each for Farm 1–3. OF samples (*n* = 8) were obtained from growing pigs from two selected pens of each barn, four barns per sampling event. Sampled pens were consistent across all sampling events. Finally, TS samples (*n* = 8) were collected from 8 individual gilts and/or sows for Farm 1–3 and 8 individual growing pigs selected by convenience (animals that approached personnel and were successfully snared) for Farm 4 and 5; both at fixed pre-selected locations within each farm.

All samples were submitted to the collaborators at the College of Veterinary Medicine, University of Minnesota (CVM-UMN) for PRRSV screening. In brief, viral RNA was extracted as described in Zhang et al. (26) and the viral nucleic acids were detected by a reverse-transcription quantitative PCR (RT-qPCR) targeting the open reading frame 6 (ORF6) of PRRSV (27). Samples with quantification cycles (Cq) < 37 were interpreted as positive for PRRSV (28, 29).

### PRRSV ORF5 Gene Sequencing, Phylogenetic Analysis, and Lineage Classification

A selection of RNA extracted PRRSV samples positive for ORF6 RT-qPCR were submitted to University of Minnesota Veterinary Diagnostic Laboratory (UMN-VDL) for sequencing of the ORF5 region. This selection was based on budget restrictions, with lowest Cq values being selected. Submitted samples were first verified for the ORF5 regions using a commercial RT-qPCR kit (OneStep RT-qPCR kit, Qiagen®, Germantown, MD). Primers and probes were not reported due to concerns in confidentiality, and samples with Cq value < 40 were classified as PRRSV ORF5 positive. Prior to the Sanger sequencing, the complete ORF5 regions in positive samples were amplified using a gel-based RT-PCR, visualized in the QIAxcel advanced system (Qiagen®, Germantown, MD), and purification using the ExoSAP-IT™ Express PCR Product Cleanup Reagent (Thermo Fisher Scientific, Waltham, MA). The purified products were submitted to University of Minnesota Genomics Center for Sanger sequencing. The raw sequences were analyzed using the SeqMan program in the Lasergene software (DNASTAR Inc., Madison, WI) for identifying mixed peaks and ruling out multiple strains. Using the MUSCLE (MUltiple Sequence Comparison by Log-Expectation) algorithm (30), the resulting sequences were aligned to 690 ORF5 reference sequences of typical PRRSV type 2 species that served as anchors for PRRSV ORF5 lineage classification previously (21, 31). In particular, 690 anchors consisted of lineages L1A (*n* = 50), L1B (*n* = 50), L1C (*n* = 50), L1D-alpha (*n* = 50), L1D-beta (*n* = 45), L1E (*n* = 50), L1F (*n* = 50), L1G (*n* = 50), L1H (*n* = 50), L2 (*n* = 28), L4 (*n* = 2), L5 (*n* = 50), L6 (*n* = 50), L7 (*n* = 15), L8 (*n* = 50), L9 (*n* = 50). In addition, ORF5 regions of five common PRRSV vaccine strains, Ingelvac PRRSV ATP (lineage L8; GenBank#: DQ988080), Ingelvac PRRSV MLV (L5; AF066183), Fostera PRRSV (L8; KP300938), Prime Pac PRRSV RR (L7; DQ779791), and Prevacent (L1; KU131568), were included. The single-nucleotide polymorphism (SNP) change, ORF prediction, and degree of identity among sequences at nucleotide were determined using Geneious Prime® (version 2021.0.3). Default penalization in profiled sum-of-pairs score for internal and terminal gaps in partial sequences was performed as described in Edgar (32).

For phylogenetic analysis, the type of PRRSV were first determined by aligning identified sequences to PRRSV type 1 (Lelystad; NC_043487.1) and 2 (VR-2332; GenBank ID EF536003.1) prototypes (21). Secondly, type 2 sequences were further aligned with 690 ORF5 sequences anchoring 16 PRRSV type 2 lineages and sub-lineages (21, 31). Maximum likelihood consensus trees were inferred using a general time reversible (GTR) model of nucleotide substitution with Γ distribution rate variation among nucleotides via the RAxML plugin (Randomized Axelerated Maximum Likelihood; version 8.2.11) in Geneious Prime® (33). Consensus support (%) was generated via bootstrap resampling with 100 iterations and the support threshold was set at 50%. Two sequences were considered identical if the nucleic acid identity (%) was over 98% (34).

### Statistical Analysis

The PRRSV detection for each specimen was described using the percentage of positives among all samples collected on the sampling event. The correlation between production data and RT-qPCR positive percentages were examined using Spearman's rank correlation coefficient (Spearman's rho) using R 4.0.4 (35). Statistical analysis was not performed for Farms 4 and 5 because of limited access to the production data, and therefore presented as descriptive statistics.

## Results

**Figure 1:**
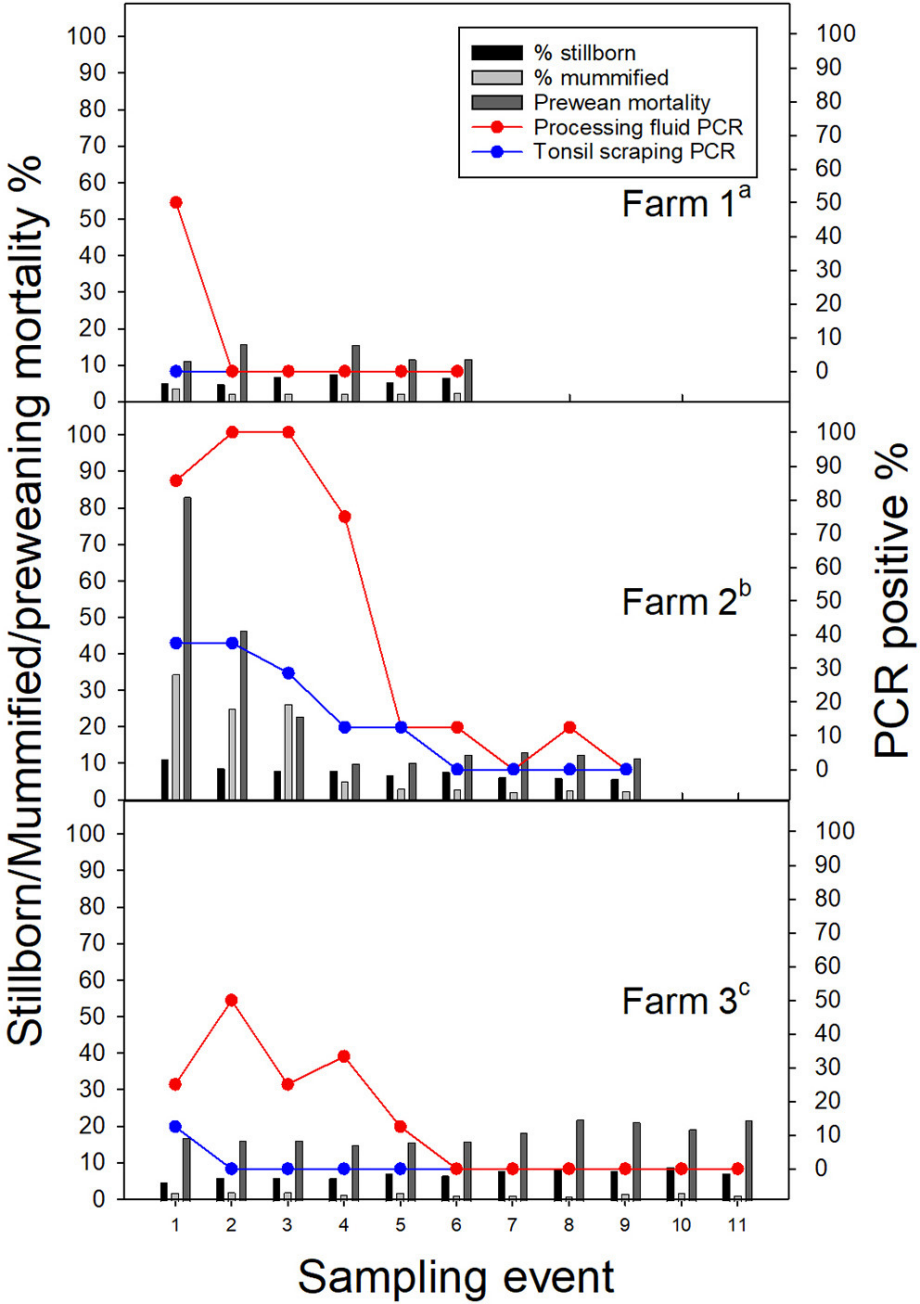
Porcine reproductive and respiratory syndrome virus (PRRSV) positive rate (%) in processing fluid and tonsil scraping specimens by stillborn fetus (%), mummified fetus (%), and pre-weaning pig death rates (%) in three breeding farms. ^a^Farm 1. Latest PRRSV outbreak, ~6 months prior to the first sampling event. ^b^Farm 2. Latest PRRSV outbreak, <1 month prior to the first sampling event. Vaccinated with a modified live vaccine (Fostera® PRRS, Zoetis Inc). ^c^Farm 3. Vaccinated with a modified live vaccine (Ingelvac PRRS® MLV, Boehringer Ingelheim).

**Figure 2:**
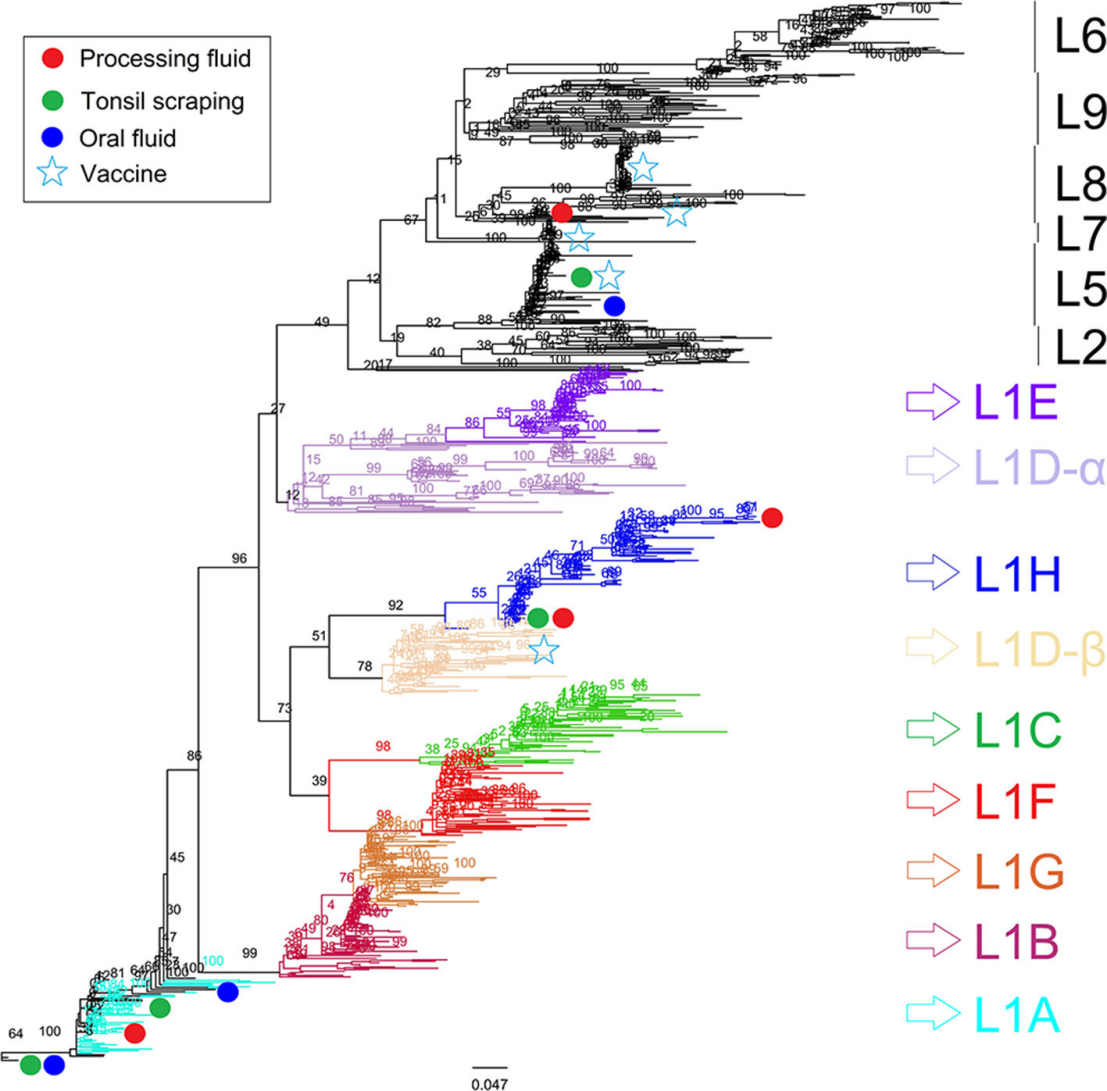
Porcine reproductive and respiratory syndrome virus (PRRSV) ORF5 phylogenetic consensus tree constructed using the maximum likelihood method with bootstrap resampling. Sequences from processing fluids (*n* = 34) of three breeding farms (Farm 1, 2, and 3), oral fluids (*n* = 7) of two growing farms (Farm 4 and 5), tonsil scraping (*n* = 12) of all recruited farms (Farms 1–5), 690 previously reported lineage anchors, and five PRRSV vaccine strains, were classified and compared. Sequences that are highly clustered are labeled only once due to the space limitation.

Among 960 subject-to-collect samples, 13 sampling events (6 in Farm 1, 3 in Farm 2, 2 in Farm 4, and 2 in Farm 5) were not carried out due to logistical challenges (Table 1). Among executed events, 30 samples (10 OF and 17 TS) were missing and 40 (25 OF and 15 TS) contained insufficient volume for testing. Thus, the final sample bank consisted of a total of 685 samples including 216 PF, 125 OF, and 344 TS. PRRSV was detected by ORF6 RT-qPCR in 20.8% processing fluids (45/216), 22.4% oral fluids (28/125), and 24.7% tonsil scrapings (85/344) (Figures 1, 2). PRRSV ORF5 sequences were successfully obtained from 34 PF (75.6% considering 45 total submitted for sequencing), 7 OF (100.0%), and 12 TS (24.5% considering 49 total submitted for sequencing) samples (Figure 2). Cq values of ORF5 RT-qPCR ranged from 21.9 to 32.8, and nucleotide length ranged from 150 to 603 base pairs (Supplementary Material 1). When excluding negative ORF5 RT-qPCR Cq values (Cq ≥ 40), samples for which ORF5 sequence could not be obtained had Cq values between 28.7 and 38.8.

**Table 1:**
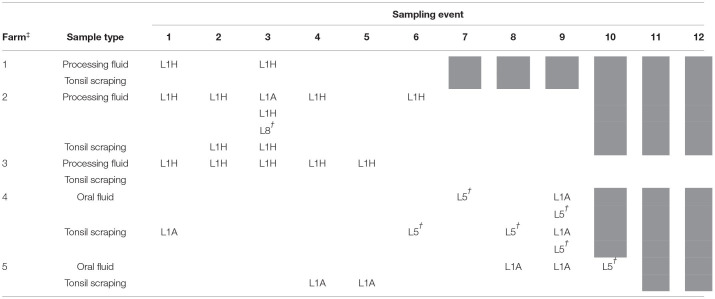
Lineages of Porcine reproductive and respiratory syndrome virus (PRRSV) open reading frame 5 (ORF5) sequences isolated overtime.

† *Sequences > 98% identical to L8 (Fostera® PRRS; Zoetis Inc.) and L5 (Ingelvac PRRS® MLV, Boehringer Ingelheim) vaccine strains.*

‡ *Farm 1: Latest PRRSV outbreak ~6 months prior to the first sampling event; Farm 2: Latest PRRSV outbreak <1 month prior to the first sampling event and vaccinated with a modified live vaccine (Fostera® PRRS, Zoetis Inc); Farm 3: Vaccinated with a modified live vaccine (Ingelvac PRRS® MLV, Boehringer Ingelheim); Farm 4: Latest PRRSV outbreak <1 month prior to the first sampling event; Farm 5: Vaccinated with a modified live vaccine (Ingelvac PRRS® MLV, Boehringer Ingelheim). Samples were not collected on grey shaded sampling events*.

All ORF5 sequences were obtained from different samples (each sample was only sequenced once) and classified into PRRSV type 2 species. Despite positive PF samples during the first sampling period, there was low PRRSV sequencing success and only one lineage detected (lineage L1H) and no significant production impacts were observed in Farm 1; which is reasonable given the herd was not knowingly exposed to PRRSV during the observation period (Figure 1). In Farm 2, high PRRSV detection along with high pre-weaning mortality and high percentage of mummified fetuses were observed in the first three sampling events, which was expected given animals were vaccinated and experiencing a recent outbreak. The RT-qPCR positivity in PF and TS samples was positively correlated to the percentage of stillborn (PF: rho = 0.86, *p* = 0.003; TS: rho = 0.89; *p* = 0.001) and mummified fetuses (PF: rho = 0.92, *p* < 0.001; TS: rho = 0.92; *p* < 0.001), respectively. In addition, three different lineages namely L1A, L1H, and L8 were identified in processing fluid samples collected on the third sampling event, indicating the simultaneous circulation of wild and vaccine strains in the herd (Table 1). The coexistence of L1A, L1H, and L5 lineages might play an important role in high PRRSV detection and poor reproductive performance in Farm 2 (Figure 1). In contrast, moderate to low viral detection and production impacts over time in Farm 3 validated a positive PRRSV status (Figure 1). Significant correlations were identified between RT-qPCR positive percentage of PF samples and all three breeding indices. In particular, RT-qPCR positivity was positively correlated with the percentage of mummified fetus (rho = 0.63, *p* = 0.04), but had negative associations with the percentage of stillborn fetus (rho = −0.82, *p* = 0.002) and pre-weaning mortality (rho = −0.65, *p* = 0.03). The counter-intuitive findings for the last two production parameters may be explained by co-circulation of other pathogens that were not investigated in the breeding herd; or the fact that the vaccine virus was being detected and contributing to the positivity, and posing a protective effect to the herd. this was a stable PRRSV farm. Most importantly, the ORF5 sequences did not cluster into the same lineage as vaccine strains, which indicated to an unexpected circulation of wild viruses in Farm 3 (Table 1).

For growing pig herds, in Farm 4, although the second batch had higher PRRSV detection in OF and TS samples, higher wean-to-finish mortality and lower ADG were reported for the first batch of animals in all four barns (Table 2). In contrast, since Farm 5 raised pigs at a later stage (finisher), similar mortality and ADG were shown even with high PRRSV detection in TS samples. Furthermore, field (lineage L1A) and modified live vaccine (lineage L5) strains of PRRSV were identified in both Farm 4 and 5 and simultaneously detected in OF and TS samples from the 9th sampling event in Farm 4 (Tables 1, 2). It is worth noting that Farm 4 was not actively vaccinated at sampling or during its recent acute outbreak, and the detection of L5 vaccine strain was not expected.

**Table 2:** Porcine reproductive and respiratory syndrome virus (PRRSV)-positive proportion (%) in oral fluid and tonsil scraping specimens, average daily weight gain, and mortalities in two production batches of two growing pig farms (Farms 4 and 5).

			**PCR positive proportion (%)**			
**Farm**	**Barn**	**Production batch**	**Oral fluid**	**Tonsil scraping**	**Mortality (%)**	**Average daily weight gain (kg)**
4^a^	1	1	10.00	50.00	19.00	1.66
		2	60.00	50.00	8.00	1.68
	2	1	0.00	55.56	12.00	1.62
		2	25.00	33.33	7.00	1.84
	3	1	11.11	66.67	6.00	1.68
		2	42.86	100.00	12.00	1.85
	4	1	0.00	70.00	7.00	1.69
		2	57.14	83.33	10.00	1.77
5^b^	1	1	57.14	66.67	4.09	1.90
		2	57.14	14.29	4.68	1.87
	2	1	10.00	53.85	4.36	1.82
		2	42.86	37.50	3.44	1.67
	3	1	14.29	66.67	6.56	1.74
		2	16.67	25.00	3.81	1.83
	4	1	0.00	87.50	5.95	1.73
		2	50.00	50.00	3.47	1.80

a *Farm 4. Latest PRRSV outbreak, <1 month prior to the first sampling event. Production batch 1 occurred April–September 2019 and production batch 2 October 2019–February 2020.*

b *Farm 5. Vaccinated with a modified live vaccine (Ingelvac PRRS® MLV, Boehringer Ingelheim). Production batch 1 occurred April–October 2019 and production batch 2 November 2019–March 2020*.

## Discussion

Under field conditions, we were able to detect up to three different PRRSV lineages (L1A, L1H, L8) within a single sampling event for a breeding herd (Farm 2), and up to two different PRRSV lineages (L1A and L5) for two growing herds (Farm 4 and 5). This was one of the first field studies to conduct ORF5 sequencing and lineage classification in multiple samples and specimen types using herds with different demographics, production stages, and PRRSV infection statuses overtime. Our findings highlighted the importance of sequencing multiple samples within a herd when conducting PRRS outbreak investigations or making important herd health decisions. Further, it also shows the deficiency and potential bias in the common practice identifying the sequences of dominant PRRSV in the field, i.e., simply submitting samples for sequencing that have the highest viral concentrations (lowest Cq).

The coexistence of multiple PRRSV genetic variants within farms and over time has been previously explored using ORF5 gene sequencing (22–24, 36). A previous study reported that 78% of the herds with multiple laboratory submissions were identified with different strains often within a 1-year period (23). In addition, multiple PRRSV variants could exist simultaneously on farms and even within an individual animal during natural infection (24). The coexistence of three genetically diverse groups of PRRSV, with ORF5 % nucleotide differences ranging from 5.8 to 11%, was reported in a chronically infected sow farm over a period of 1 year (36). Besides, because of inconsistent cross-protection, co-circulation of PRRSV variants may cause clinical losses in production systems (37), challenge the efficacy of vaccination, and often introduce additional complexity in disease control (38–40).

Although PF, OF, and TS have been reported as promising specimens for PRRSV field diagnostic (5), results yielded from the different sample types should not be directly compared due to the inherent differences in sample collection and potentially sensitivity levels. As such, discrepancies in RT-qPCR results among PF, OF, and TS (Figure 1 and Table 2) could be attributed to the fact that aggregated PF and pen-based OF samples were collected from multiple litters/animals, while TS samples were collected from individual animals. Furthermore, the sample quality and quantity might be associated with the differences in PCR positivity between the sample types. Investigating these factors were outside of the scope of the study, which was focused on implementing sample collections that would be commonly used under field conditions.

Although only representing ~4% of the whole PRRSV RNA genome (20), the ORF5 gene is a highly diverse region of PRRSV and has been widely used for PRRSV determination and lineage classification via genetic sequencing and phylogenetic analyses, respectively (5). A recent study (41) classified 102 PRRSV ORF5 sequences obtained from 18 U.S. states (2014–2018) along with 84 GenBank references and suggested the majority of ORF5 sequences belonged to the lineage 1 (L1), which is the same lineage as the MN-184, NADC30, and the Prevacent® vaccine. In contrast, Oklahoma and Illinois were dominated by lineage 5 (L5) sequences. In the current study, except for five samples identical to the Ingelvac® and Fostera® vaccine strains (L5 and L8), ORF5 sequences were classified in the sub-lineages L1A and L1H, which was consistent with a recent study (21) that identified a transition of dominant lineages from L9 to L1 using over 4,000 ORF5 sequences isolated from U.S. swine herds since 2014. The emerging L1A and L1H sub-lineages have been identified in swine diagnostic samples from 38 U.S. states with detection increased by 30.54% and 12.81%, respectively, since 2013 (42). Given the farm vaccination and infection statuses, the detection of field isolates (L1A and L1H) in Farm 1, 3, and 5 might be the consequence of previous outbreaks, as PRRSV could circulate within swine herds for long periods of time, even when implementing control and elimination strategies (19). Likewise, the L5 vaccine strain identified in Farm 4 might be from previous vaccination, although this information could not be obtained. Lastly, the low to absent detection of vaccine isolates in Farm 3 and 5 was unexpected but could be explained by the low successful rate of ORF5 sequencing, or simply low levels of attenuate vaccine-type virus shedding in the herd.

One of the limitations of our study was the small number of ORF5 sequences recovered, especially for OF and TS specimens. In addition, despite the critical role of ORF5 sequencing and phylogenetic analysis in PRRSV diagnosis, their viability in OF samples has been rarely explored. As reported by Zhang et al. (26), PRRSV ORF5 sequencing using the traditional Sanger sequencing method was accomplished in 95% OF samples with Cq < 31, and the success rate declined in samples with higher Cq. Similarly, the full-length genome sequencing was only achieved in OF samples with Cq ≤ 20.6. Consistently, the current study obtained ORF5 sequences in OF samples with Cq of ORF5 RT-qPCR ranging between 27.6 and 30.5 although only seven OF samples were sequenced. This may reveal issues in the efficiency and reliability of PRRSV genome sequencing using OF samples collected under field conditions, considering OF often contains lower concentration of viral nucleic acids than serum (43, 44). Likewise, the low success of ORF5 sequencing in TS samples suggested they may not be a promising specimen for these purposes. Notably, even though PF sequencing has become common in the past years, its nature as an aggregate specimen, i.e., yielded from multiple animals within and across litters, may partially explain the diversity observed. Further research focusing on sequencing of individual piglets and comparisons between those sequences with sequences obtained from composite samples across piglets of the same litters and across litters are warranted. Furthermore, the present study did not investigate recombination events among obtained sequences, anchors, and vaccine strains as this falls outside of the primary objective, although it is without doubt that PRRSV diversity can be attributed to recombination among wild and vaccine strains (45).

Performance impacts by PRRSV infections have challenged the swine industry over decades and has been one of the major motivations driving routine PRRSV detection and control in breeding herds. In particular, the increased number of mummified fetuses and piglet pre-weaning mortality have previously been reported not only for swine experiencing wild-type PRRSV infections, but also for naïve swine exposed to MLV PRRSV vaccines (46). Consistently, the current study observed moderately positive correlations between PRRSV detections and reproductive performance in a breeding herd with recent PRRSV outbreak (Farm 2). Since MLV vaccination may influence the reproductive performance, a common question raised by swine veterinarians is whether observed PRRSV clinical signs are caused by a vaccine or wild-type PRRSV. In our study, despite the one-time detection, the coexistence of multiple PRRSV lineages/sub-lineages (L1A, L1H, and L8) might have played a role in high PRRSV detection and poor reproductive performance in Farm 2 (Figure 1). However, the association between PRRSV lineages and virulence could not be determined by this study given the low recoverability of ORF5 sequences and lack of accompanying production data. Thus, this knowledge gap must be fulfilled by further research.

In conclusion, the data reported herein demonstrated the presence of multiple PRRSV strains, including vaccine and wild viruses, among and within sampling events in commercial breeding and growing swine herds over time. Furthermore, the phylogenetic analysis and lineage classification suggested the potential bias and inappropriateness of the current diagnostic practice, i.e., determining the strain of circulating virus by sequencing a single sample with the highest concentration of PCR targets. This study also confirmed the association between PRRSV infection and reproductive performance in a commercial breeding herd; and generated hypothesis on the potential negative impact of co-circulation of different PRRSV lineages on production parameters; which should be a focus for future studies.

## Data Availability Statement

The original contributions presented in the study are included in the article/Supplementary Material, further inquiries can be directed to the corresponding author/s.

## Ethics Statement

Ethical review and approval was not required for the animal study because an Institutional Animal Care and Use Committee (IACUC) approval was not required for this project. All sample collections were performed by farm veterinarians for each participating farm. Written informed consent for participation was not obtained from the owners because the sample used in this project was collected by farm veterinarians as part of their routine duties. The communication between the project investigator and veterinarians was done via phone calls.

## Author Contributions

T-YC, MC, DS, SM, JF, and AA: conceptualization. T-YC, MC, DS, MY, SM, JF, and AA: methodology, data acquisition, analysis, interpretation, and writing—review and editing. T-YC, MC, and AA: writing—original draft preparation. All authors have read and agreed to the published version of the manuscript.

## Funding

This research was funded by the National Pork Board, grant number 18-167.

## Conflict of Interest

The authors declare that the research was conducted in the absence of any commercial or financial relationships that could be construed as a potential conflict of interest.

## Publisher's Note

All claims expressed in this article are solely those of the authors and do not necessarily represent those of their affiliated organizations, or those of the publisher, the editors and the reviewers. Any product that may be evaluated in this article, or claim that may be made by its manufacturer, is not guaranteed or endorsed by the publisher.

## Supplementary Material

Supplementary Material 1 PRRSV ORF5 sequences and the identity matrix including 690 anchors of nine L1 sublineages (L1A – L1C, L1D-alpha, L1D-beta, and L1E – L1H) and seven lineages (L2 and L4 – L9), and ORF 5 sequences of five common vaccine strains (Ingelvac PRRSV ATP [lineage L8; GenBank#: DQ988080], Ingelvac PRRSV MLV [L5; AF066183], Fostera PRRSV [L8; KP300938], Prime Pac PRRSV RR [L7; DQ779791], and Prevacent [L1; KU131568]).
